# Coordination of COVID-19 platform trials in Europe

**DOI:** 10.1186/s13063-024-08126-5

**Published:** 2024-04-25

**Authors:** Jacques Demotes, Victoria Charlotte Simensen, Keiko Ueda, Sareema Javaid, Paula Garcia, Burç Aydin, John-Arne Røttingen

**Affiliations:** 1 European Clinical Research Infrastructure Network, ECRIN, 30 Boulevard Saint-Jacques, Paris, 75014 France; 2 Department of Vaccines and Immunisation, Division of Infectious Disease Control, Norwegian Institute of Public Health, Oslo, Norway; 3 Division of Infectious Disease Control, Norwegian Institute of Public Health, Oslo, Norway

**Keywords:** Adaptive platform trial, COVID-19, Coordination mechanism

## Abstract

To ensure optimal coordination of the EU-funded COVID-19 platform trials, a double coordination mechanism was established. It included the Trial Coordination Board (TCB) to promote the dialogue between investigators and relevant public health stakeholders and the Joint Access Advisory Mechanism (JAAM) to streamline access of new intervention arms to the platform trials. Both the TCB and the JAAM emerged as efficient instruments to promote cooperation and optimise the use of resources within EU-funded adaptive platform trials. In addition, an adaptive platform trial toolbox was developed to collect information and literature on challenges and solutions identified to date. The recently funded ‘Coordination MEchanism for Cohorts and Trials’ (CoMeCT) project will endeavour to make this model sustainable, with a further expansion to other emerging infectious diseases, as part of the governance of the current and future platform trials for pandemic preparedness. This example could serve as a model for platform trial coordination in other disease areas.

## Background

On March 11, 2020, the World Health Organization (WHO) declared the coronavirus disease 2019 (COVID-19) as a global pandemic and called for urgent and powerful action. The European Medicines Agency (EMA) rapidly called for large, multi-arm clinical trials, and the European Union (EU) responded with dedicated funding calls under research and innovation programmes to support clinical study operations and infrastructure in the fight against COVID-19 [1–3]. A key element of this effort has been designing and conducting large adaptive platform trials (APTs) as a public health measure to rapidly identify effective, safe, and scalable therapeutic options in affected populations, addressing either repurposed or innovative solutions.

**Fig. 1:**
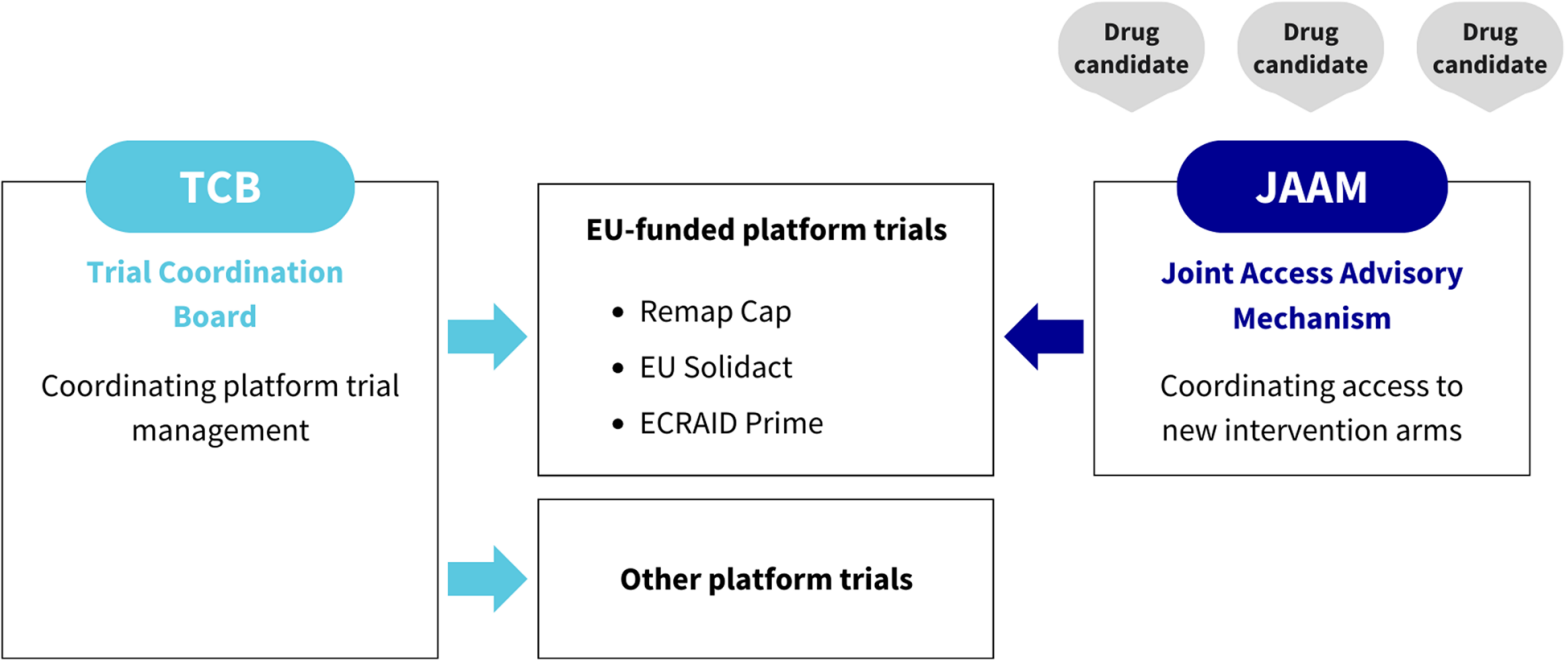
The trial coordination board (TCB) promotes cooperation between the platform trials (both EU-funded or not EU-funded) and establishes a dialogue with policymakers, while the Joint Access Advisory Mechanism (JAAM) acts as an access board conducting independent assessment of the public health value of individual intervention arms and making recommendations on the most suitable EU-funded platform trial to test a drug candidate

Additionally, optimal use of such research tools in the pandemic context has required efficient governance and strong coordination to avoid unnecessary competition, overlap, and duplication and to make the best possible use of the APT infrastructure, in particular with regard to the selection of new intervention arms. A common coordination module (https://covid19trials.eu/en) constructed as a shared Work Package (WP) between the projects EU RESPONSE (GA 101015736) and RECOVER (GA 101003589) was implemented in July 2020 with three distinct components. The *Trial Coordination Board (TCB)* was established to create a cooperative forum for dialogue across the large COVID-19 APTs and with the relevant stakeholders. The *Joint Access Advisory Mechanism (JAAM)* was developed to provide scientific advice on candidate interventions and recommendations on the best adapted trial design and population (Fig. 1). Lastly, the *Adaptive Platform Trial Toolbox* was created to collate resources to support the development of future APTs.

## Trial Coordination Board (TCB)

The TCB was designed as a trusted forum for dialogue between COVID-19 therapeutic APTs and major health research stakeholders, to promote coordination and dialogue. In addition to the EU-funded COVID-19 adaptive platform trials, the TCB invites global clinical research partners to participate in discussions (Table 1). Through biweekly meetings, APT investigators share early information on plans for new interventions, recruitment status, site activation, interim analyses, and future publications. This information is summarised in a dashboard circulated with the minutes to meeting participants after every meeting. Moreover, the TCB established a confidential dialogue between the trial data and safety monitoring boards to share unblinded information on safety signals, which is relevant whenever trials assess chemically related interventions or compounds acting through similar mechanisms.

**Table 1:** COVID-19 adaptive platform trials represented in the trial coordination board

COVID-19 adaptive platform trials attending regularly	
*Trial name*	*Trial identifier*
AMMURAVID	EudraCT2020-001854–23
ANTICOV	PACTR202006537901307
ACTIV-3	NCT04501978
ACTIV STRIVE	NCT05605093
DisCoVeRy^a^	EudraCT2020-000936–23
ECRAID-Prime^a^	EU GA 101046109
EU-SolidAct^a^	EudraCT2021-000541–41, EUCT2022-500385–99-00
MANTICO-2	EudraCT2021-002612–31
PRINCIPLE	EudraCT2020-001209–22
PANORAMIC	EudraCT2021-005748–31
RECOVERY	EudraCT2020-001113–21
World Health Organization SOLIDARITY	EudraCT2020-001784–88
REMAP-CAP^a^	EudraCT2015-002340–14
MPX RESPONSE^a^	Cohort
VACCELERATE^a^	EU GA 101037867, EudraCT2021-004526–29 and EudraCT2021-004889–35
COMCOV1	EudraCT: 2020–005085-33
COMCOV2	EudraCT: 2021–001275-16
COMCOV3	EudraCT: 2021–004267-27
MonkeyVax	Cohort

*APT* Adaptive platform trial

^a^European Union-funded COVID-19 adaptive platform trial

The extended TCB facilitates the dialogue of APT investigators with stakeholders, policymakers, and regulators (Table 2) to discuss advancements and hurdles experienced in APTs. Between July 2020 and October 2023, 80 virtual meetings have been held, addressing various issues around trial design, conduct, results, and the relevance of findings for clinical care (Table 3). The TCB meetings also serve as ‘networking events’, where opportunities for bilateral or multilateral collaborations arise. Such collaborations may concern specific compounds, trial arms, methods, recruitment, population-specific questions, or the need to conduct meta-analyses to better understand the evidence base and tailor practice. The TCB has also contributed to informing and exchanging on relevant preparedness and response initiatives in Europe, hence aligning and focusing research priorities. Discussions in the TCB have inspired a structured focus on subpopulations, including outpatients, through the set-up of the multinational outpatient platform trial, ECRAID-Prime (GA 101046109). This trial joined the coordination module. The TCB has taken a proactive role in advising trials for new outbreaks, i.e. with the inclusion of the mpox MPX RESPONSE project (GA 101115188). The TCB has gradually evolved the scope of its discussions to reflect the focus of its affiliated networks as these have expanded beyond COVID-19 and recruited patients on the basis of other respiratory tract viruses and on pathogen-agnostic syndromic presentation.

**Table 2:** Stakeholders participating in the trial coordination board

COVID-19 clinical research stakeholders
Coalition for Epidemic Preparedness Innovations
COVID-19 Therapeutics Accelerator
European Centre for Disease Prevention and Control
European Commission
European Federation of Pharmaceutical Industries and Associations/Vaccines Europe
European Health and Digital Executive Agency
European Medicines Agency
European Network for Health Technology Assessment
EU-PEARL Project (IMI GA 853966–2)
Heads of Medicines Agencies Clinical Trials Facilitation and Coordination Group
World Health Organization

**Table 3:** Selected examples of discussion topics

Selected discussion topics
Regular trial- and country-specific information updates: new or closing study arms, changes in inclusion/exclusion criteria, results and upcoming publications, updates in local standard of care, and drug provision challenges
Data sharing initiatives and options for the optimal sharing of data across trials and cohorts
Drug repurposing efforts across Europe and beyond (multi-stakeholder, national and regional initiatives)
Regional and international evidence synthesis initiatives for the development of guidelines
Funding calls and programmes for further COVID-19 clinical research
Opportunities for collaboration and areas of mutual interest with industry for early clinical development and APT management and regulation
Longer-term consequences of COVID-19, ongoing trials and cohorts of interest and definition of ‘long COVID’
New safety signals of interventions being assessed across the COVID-19 APTs
Correlates of protection and surrogate outcomes
Laboratory standardisation and synergies
Trials for COVID-19 in the primary care setting
Observational cohort studies (e.g. ORCHESTRA project, EU GA 101016167), post-marketing studies, and collaboration with COVID-19 APTs
Patient recruitment challenges
Regulatory submission and evaluation processes in the pandemic context, perspective of the regulatory bodies and ethics committees, transition to the Clinical Trials Information System in Europe, regulatory processes in other world regions, and opportunities for growth and mutual recognition
Scientific and ethical considerations for special populations of pregnant women, immunocompromised patients, and children

A vaccine pillar was added to the TCB in March 2022, through the involvement of the EU-funded VACCELERATE project (GA 101037867) and the expansion of the network by adding vaccine trialists and stakeholders from the broader vaccine field, such as the Coalition for Epidemic Preparedness Innovations (CEPI). Meetings have subsequently been held both with the specific ‘vaccine pillar’, the initial ‘therapeutic pillar’, or a combination of the two, a ‘Joint TCB’, addressing both preventive and therapeutic strategies. The TCB has taken a proactive role in strengthening the dialogue between researchers and policymakers on the implications of shifting epidemiology and guidelines and their impact on recruitment to vaccine trials.

The TCB aspires to become a sustainable instrument promoting optimal use of resources and a consistent pan-European research strategy in health emergencies as a problem solving, coordination, and collaboration mechanism in the global health landscape. Through the recently funded CoMeCT project (GA101136531, https://comectproject.org), the TCB will continue to expand its activities beyond the COVID-19 platform trials (both for treatments and for vaccines), also addressing other emerging infectious diseases and including the new platform trials that will be funded following the Horizon Europe (HE) call HORIZON-HLTH-2024-DISEASE-03–11 on platform trials for pandemic preparedness. The CoMeCT project also includes a similar coordination mechanism for infectious diseases cohorts, the Cohort Coordination Board (CCB), based on the mechanism already developed in the H2020 ORCHESTRA project (GA 101016167).

## Joint Access Advisory Mechanism (JAAM)

The JAAM was designed as both an access board and a coordination mechanism for the COVID-19 platform trials. The COVID-19 APTs were funded as instruments, or infrastructures, able to address multiple scientific questions, with a peer-review covering the master protocol but not the evaluation of individual intervention arms. The JAAM was established as an independent access board in charge of assessing the scientific value and medical relevance of candidate therapeutic interventions, to promote optimal use of the APT resources and of the patient populations. In addition, the JAAM plays a coordination role acting as a common access board for the series of EU-funded COVID-19 APTs, prioritising the requests from academic or industry innovators willing to test their interventions in primary care patients, in hospital patients, or in intensive care patients. The JAAM involves an independent expert panel of seven international scientists, not directly involved in the projects or the trials in scope, and the APT investigators and funders act as observers of this board. Candidate interventions submitted to the JAAM secretariat undergo scientific assessment based on the following criteria: public health interest; scientific, medical, and ethical soundness; appropriate patient population, comparator, and outcomes; and promotion of coordination and optimal use of resources. The JAAM recommendations are not binding, and the final decision on including new interventions in the EU-funded COVID-19 platform trials remains at the individual trial level. The recommendations also include provisions on the most appropriate platform trial and patient population (primary care, hospital, or intensive care patients) in which to test the candidate intervention. From February 2021 to October 2023, the JAAM received 26 requests, of which 24 were analysed by the secretariat. In total, 18 virtual meetings were conducted resulting in recommendations on 14 candidate interventions, including both innovative medicines and repurposed compounds.

The CoMeCT project will be an opportunity for the JAAM to enlarge its scope beyond COVID-19 therapeutics, also covering the newly funded platform trials for pandemic preparedness addressing other infectious diseases, and vaccines. A systematic horizon scanning on the development and repurposing pipelines against viral and bacterial infections will help attract relevant candidates for new intervention arms. This could be facilitated, for example, through a closer, more systematic exchange with entities such as the European Medicines Agency (EMA) or the Health Emergency Response Authority (HERA).

## Adaptive platform trials toolbox

In addition, an adaptive platform trial toolbox collects open-access scientific and operational resources to facilitate the design, planning, and conduct of APTs for future pandemics or other disease areas. Public access, relevance, conformity with the current regulatory and ethical framework, and practicality were the key considerations for the inclusion of the resources. The toolbox has been publicly accessible on the web since June 2021 and was recently updated in October 2023. It now includes 131 tools classified in the following categories: trial design and conduct, regulations, data management, statistics, and resources from ongoing platform trials (https://covid19trials.eu/en/adaptative-platform-trial-toolbox). Users are invited to provide contributions for the inclusion of additional resources.

## Conclusion

APTs have delivered practice-changing and life-saving evidence on multiple drugs for the management of COVID-19 [4, 5]. Yet, these complex trials are not devoid of challenges and require specific governance, coordination mechanisms, and dedicated support to promote robustness in trial design, conduct, and results in a timely manner. Along with other lessons learned from clinical research during the pandemic [4, 6, 7], it is expected that this experience will promote a culture of scientific collaboration and coordination in health research (Table 4).

**Table 4:** Generic recommendations to promote coordination and optimal use of resources in platform trials

Consider adaptive platform trials as public health research instruments
Avoid development of overlapping platform trials
Avoid competition between platform trials (in terms of sites, patient recruitment, intervention arms)
Promote coordination between platform trial managers
Build a trusted dialogue and cooperation mechanisms between investigators of platform trials addressing the same diseases
Establish an independent board providing scientific assessment and prioritising the candidate intervention arms
Use this independent board to distribute the new intervention arms to the best adapted platform trial

## Abbreviations

APT: Adaptive platform trial
CCB: Cohort coordination board
CEPI: Coalition for Epidemic Preparedness Innovations
COVID-19: Coronavirus disease 2019
EMA: European Medicines Agency
EU: European Union
GA: Grant agreement
HERA: Health Emergency Response Authority
JAAM: Joint Access Advisory Mechanism
TCB: Trial Coordination Board
WHO: World Health Organization
WP: Work Package
